# Efficacy, safety, and tolerability of adjunctive Lacosamide therapy for focal seizures in young children aged ≥1 month to ≤4 years: A real‐world study

**DOI:** 10.1111/cns.14917

**Published:** 2024-08-09

**Authors:** Lu Yang, Yuhang Liu, Yu Deng, Xiaoling Peng, Qiao Hu, Li Jiang, Yue Hu

**Affiliations:** ^1^ Department of Neurology Children's Hospital of Chongqing Medical University, National Clinical Research Center for Child Health and Disorders, Ministry of Education Key Laboratory of Child Development and Disorders, China International Science and Technology Cooperation base of Child development and Critical Disorders, Chongqing Key Laboratory of Child Neurodevelopment and Cognitive Disorders, Big Data Engineering Center Children's Hospital of Chongqing Medical University Chongqing China; ^2^ Guangdong Provincial Key Laboratory of Interdisciplinary Research and Application for Data Science BNU‐HKBU United International College Zhuhai China

**Keywords:** adjunctive therapy, children, focal seizures, lacosamide

## Abstract

**Aims:**

To evaluate the efficacy, safety, and tolerability of adjunctive lacosamide therapy against focal seizures in young children (1 month – 4 years).

**Methods:**

This non‐randomized, open‐label, and self‐controlled real‐world study included 105 children (1 month–4 years) with focal seizures treated with adjunctive lacosamide therapy at Children's Hospital of Chongqing Medical University.

**Results:**

(1) The 50% response rates at 3, 6, 9, and 12 months of follow‐up were 58.1%, 61.0%, 57.1%, and 56.2%, while the seizure‐free rates were 27.6%, 34.3%, 32.4%, and 37.1%, respectively. The 50% response rate of the first addition of lacosamide for focal seizures was much higher than the second and later added treatment at 3 months (*p* = 0.038). After 1 year of follow‐up, these children showed an improvement in neurodevelopmental levels (*p* < 0.05). (2) Lacosamide retention rate was 72.7% (64/88) after 1 year of follow‐up. Lack of efficacy and serious adverse events were independent risk factors for the lacosamide retention rate. (3) During adjunctive lacosamide therapy, 13 (12.4%) patients reported adverse events and five (4.7%) patients withdrew due to adverse events, including vomiting drowsiness, ataxia (0.94%), neck itching with eczema (0.94%), irritability (1.88%), and gastrointestinal discomfort (0.94%).

**Conclusion:**

Adjunctive lacosamide therapy was effective, safe, and well‐tolerated in young Chinese children with focal seizures in this study.

## Graphical abstract

Lacosamide (LCM) is effective, safe, and well‐tolerated as an adjunctive therapy in young Chinese children aged ≥1 month to ≤4 years. The order of lacosamide introduction significantly influenced its short‐term efficacy.

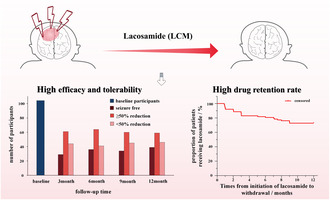

## 1 INTRODUCTION

The third‐generation antiseizure medication (ASM) lacosamide (LCM), the R‐enantiomer of 2‐acetamido‐N‐benzyl‐3‐methoxypropionamide, is a novel functionalized amino acid. The therapeutic mechanisms of LCM can be summarized as follows: (1) LCM appears to selectively enhance the slow inactivation of voltage‐gated sodium channels without enhancing their fast inactivation, thereby stabilizing the neuronal membrane, reducing the continuous excitability of neurons, and effectively controlling epileptic seizures without affecting normal physiological function.^1^ (2) LCM can inhibit collapsin response regulatory protein 2 (CRMP2)‐mediated microtubulin polymerization and astrocyte semi‐channel function, thereby reducing abnormal synaptic recombination in the brain,^2^ reducing the release of ATP and glutamate from astrocytes, and thus playing an antiepileptic effect.^3^ (3) GABA is an inhibitory neurotransmitter, and GABA receptors become unstable under the continuous neurotransmitter release during seizures. LCM targets GABA receptors and, when combined with levetiracetam, can improve cortical γ‐aminobutyric acid damage and restore GABA function in patients with epilepsy.^4^ LCM can be administered through oral and intravenous routes, and has the characteristics of good pharmacokinetics, small protein structure, high oral bioavailability, a short half‐life (13 h), low drug interactions, and good safety.^5^ Its ability to easily penetrate the blood–brain barrier allows the concentration of LCM in the cerebrospinal fluid to reach approximately 85% of that in serum.^6^ The good pharmacokinetics of LCM in children aged 4–17 years are similar to that in adults, with 100% oral bioavailability, below 15% plasma protein binding, and relatively few drug interactions.^7^, ^8^, ^9^

In 2008, the United States Food and Drug Administration (FDA) approved LCM as an adjunctive treatment for focal seizures with or without secondary generalized tonic–clonic seizures in adults and adolescents over 16 years.^10^ Since then, several randomized, double‐blind, large‐scale, multicenter clinical trials evaluating both LCM monotherapy and adjunctive therapy have been conducted.^10^, ^11^, ^12^, ^13^ On the basis of the efficacy data from adult studies and the available pharmacokinetic and safety data in children, LCM was approved by the FDA in 2014 and the European Medicines Agency in 2016; these approvals were granted for the use of LCM in both monotherapy and adjunctive treatment for focal epilepsy in patients aged 4 years and older.^14^ LCM can effectively reduce the frequency of seizures in patients aged 4–17 years with focal seizures.^15^ In October 2021, the FDA expanded the approval of LCM to include its use in patients older than 1 month with focal seizures.^16^ In August 2019, the China National Medical Products Administration granted approval for adjunctive LCM treatment for focal seizures in children aged 4 years and older. However, evidence regarding the efficacy, safety, and tolerability of LCM in young children with epilepsy aged between 1 month and 4 years is limited. At present, the treatment of pediatric epilepsy is constrained by numerous challenges, particularly those related to conducting clinical drug trials and research involving younger children with epilepsy. In this scenario, real‐world studies (RWS) have emerged as an important complement to clinical research in the field of early childhood epilepsy.

In this manuscript, we report the findings of a non‐randomized, open‐label, self‐controlled RWS without a placebo conducted at Children's Hospital of Chongqing Medical University in which 105 young children (≥1 month–≤4 years) with uncontrolled focal seizures were treated with adjunctive LCM therapy from January 1, 2020, to May 31, 2023. The purpose of this study was to analyze the efficacy, safety, and tolerability of adjunctive LCM therapy in young children with focal seizures, explore the factors that may influence its effectiveness, and provide guidance for the clinical application of this therapy in this specific population.

## 2 METHODS

### 2.1 Participants

#### 2.1.1 Inclusion criteria

1. Patients treated with adjunctive lacosamide therapy at Children's Hospital of Chongqing Medical University from January 1, 2020, to May 31, 2023
2. Children aged ≥1 month to ≤4 years
3. Patients who met the International League Against Epilepsy (ILAE) diagnostic criteria for epilepsy proposed in 2017.^17^ Specifically, all patients had focal seizures, which may or may not have progressed to secondary bilateral tonic–clonic seizures
4. Patients with a history of using one or more ASMs without adequate seizure control with the previous medication(s)
5. The study protocol was approved by the Ethics Committee of Children's Hospital Affiliated to Chongqing Medical University [Approval document No. (2023) ethics review (research) No. 474]. Informed consent was obtained from the children's guardians.

#### 2.1.2 Exclusion criteria

1. Patients with a documented allergy or severe intolerance to the active ingredient of LCM or any excipients present in the drug
2. All patients routinely completed the electrocardiogram (ECG) before premedication. Patients who exhibited a second‐degree or third‐degree atrioventricular block as detected by ECG.

### 2.2 Design and measures

LCM from UCB Pharma S.A (Registration Standard: JX20110253) was administered as adjunctive therapy without altering or replacing the patient's existing ASM regimen. Before initiating adjunctive LCM therapy, the patient's guardians were provided with a detailed explanation of the efficacy and potential adverse events of the drug. The initial oral dose of LCM was 2 mg/kg/day, with 2 mg/kg increments every week. The goal was to reach a target maintenance dose of 6–12 mg/kg/day, which was divided into two doses throughout the day. The medication dose could be adjusted based on the patient's actual clinical presentation.

Efficacy evaluation: The mean number of monthly seizures during the 3‐month period before LCM treatment was set as the baseline seizure frequency (“no. of seizures/month”). The percentage rate of seizure reduction was defined as: (baseline frequency‐post‐treatment frequency)/baseline frequency * 100%. Evaluation of efficacy was categorized as follows: the seizure‐free rate means a 100% reduction in seizure frequency; the effective rate (50% response rate) refers to at least a 50% reduction in seizure frequency; the ineffective rate (lack of efficacy) is a seizure frequency reduction of less than 50% and/or negative effect on seizure pattern (increase in seizure severity or frequency).^13^, ^18^

We adopted a self‐controlled design and collected the frequency of included children's seizures before adjunctive LCM therapy and 3, 6, 9, and 12 months after adjunctive LCM therapy. “Self‐controlled” is a comparison of the effects of different measures on the subject himself at the two stages before and after, to exclude individual differences as much as possible, and to obtain real conclusions. The seizure frequency before LCM addition was used as the baseline seizure frequency. The frequency of seizures after 3 months (short‐term efficacy), 6 months, 9 months, and 12 months (long‐term efficacy) of adjunctive LCM treatment was compared with the baseline seizure frequency.^19^ If other treatments, including ketogenic diet, epilepsy surgery, and vagus nerve stimulation, were added or the ASM was replaced, the efficacy data were withdrawn from observation, but safety was still evaluated.

Neurobehavioral development was assessed using the Gesell Developmental Schedules (GDS) developed by the American Gale Research Association. The time points for assessment were baseline and 1 year after the addition of LCM. The development quotient (DQ) for social, adaptive, gross motor, fine motor, and language domains was used to evaluate neurodevelopmental performances (DQ = developmental age/chronological age ×100). DQ ≥85 was classified as typically developing, while 75 ≤ DQ < 85 was considered borderline. DQ < 75 indicated delayed.^20^, ^21^

The definition of drug‐resistant epilepsy was based on the guidelines of the ILAE. Thus, drug‐resistant epilepsy means failure of adequate trials of two tolerated, appropriately chosen, and used antiseizure medication schedules (whether as monotherapies or in combination) to achieve sustained seizure freedom.^22^

Cranial magnetic resonance imaging (MRI) was evaluated by a professional radiologist. Cranial MRI abnormalities mainly included encephalomalacia (①low signal on T1‐weighted images, ②high signal on T2‐weighted images, ③low signal on FLAIR images, surrounded by a high signal area, and clear boundary.) and/or brain atrophy, neuronal migration disorder or hippocampal sclerosis. Relevant diagnostic criteria are used as described in reference.^23^, ^24^, ^25^

The observation period ended on December 31, 2023.

### 2.3 Data analysis

Statistical analysis of the data was performed using SPSS version 25.0 (IBM Corporation) and SAS version 9.4 (SAS Institute). Count data were described by frequency and constituent ratio (missing data were excluded), and categorical variables were described by percentage (%). The normality of measurement data was judged by Shapiro–Wilk test. Data were considered to fit within a normal distribution when *p* > 0.05. Measurement data with a normal distribution were described as mean ± standard deviation (x ± s), and measurement data with a non‐normal distribution were described as median and interquartile range. One‐way analysis of variance was used for comparisons among multiple groups. The least significant difference method was used for pairwise comparisons among multiple groups when the variance was equal, and the Tamhane test was used in cases with uneven variance; *p* < 0.05 was considered statistically significant.

Patients dropped out due to lack of efficacy, adverse events, and other factors (i.e., changes in the treatment plan or withdrawal of LCM). The missing mechanism of these missing data was categorized as missing not at random (MNAR),^26^ while the missing mode was categorized as monotone missing mode. We used multiple imputations to handle missing data through treatment strategies, composite strategies, and hypothetical strategies. The Mixed Model for Repeated Measures (MMRM) analysis method was used to analyze the sensitivity of the data before and after imputation.^27^ The fixed effects were group (group before imputation, group after imputation), time, and the interaction term (group * time).

## 3 RESULTS

### 3.1 Clinical features and auxiliary examinations

This study included 105 young children with focal seizures aged ≥1 month to ≤4 years who visited Children's Hospital of Chongqing Medical University from January 1, 2020, to May 31, 2023, and received adjunctive LCM therapy (Table 1).

**TABLE 1:** Risk factors affecting short‐term (3 months) efficacy of adjunctive LCM therapy for focal seizures in children aged 1 month to 4 years.

Characteristic	Total (*n* = 105)	Effective (*n* = 55)	Ineffective (*n* = 37)	Z/X^2^	*p*
Average age, years	2.18 (1.04,3.02)	1.87 (0.80,2.07)	2.33 ± 0.12	−1.684	0.092
Sex					
Female	46 (43.8%)	23 (41.8%)	18 (48.6%)	0.418	0.518
Male	59 (56.2%)	32 (58.2%)	19 (51.4%)		
Weight, kg	12 (9,14)	12 (8.5,14)	13 (9.3,15)	−1.265	0.206
Developmental delay	70 (66.7%)	31 (56.4%)	27 (73%)	2.619	0.106
GDS DQ scores					
Social domain	51.65 (40.78,67.43)	49.03 ± 3.54	57.1 (43,61)	−0.6	0.548
Adaptive domain	52.06 ± 18.09	51.45 ± 3.39	52.08 ± 3.32	−0.249	0.803
Gross motor domain	56.75 (39.58,69.93)	50.43 ± 3.86	55.43 ± 3.2	−0.920	0.358
Fine motor domain	53.00 (34.13,67.93)	49.93 ± 3.74	53.53 ± 3.22	−0.787	0.431
Language domain	53.50 (37.38,67.00)	48.92 ± 3.36	52.21 ± 3.59	−0.772	0.440
Family history of epilepsy	16 (15.2%)	10 (18.2%)	6 (16.2%)	0.059	0.807
Asphyxia at birth	11 (10.5%)	7 (12.7%)	3 (8.1%)	0.127	0.722
Complications during pregnancy	8 (7.6%)	3 (5.5%)	3 (8.1%)	0.006	0.940
Mean duration of seizures, months	8 (3,18.5)	6 (2,12)	12 (6.5,23)	−3.238	0.001
Mean age at seizure, months	10.33 (5.32,20.50)	9.9 (3.76,22.13)	10.52 (5.6,19.89)	−0.187	0.852
Frequency of seizures, episodes/month	9 (3,73.5)	8 (3,54)	21 (1.5,137)	−0.791	0.429
Duration of a seizure, s	60 (10,172.5)	60 (10,90)	90 (10,240)	−1.321	0.187
Classification of seizure types				7.863	0.049
Focal motor seizures	58 (55.2%)	32 (58.2%)	20 (54.1%)		
Focal non‐motor seizures	4 (3.8%)	3 (5.5%)	1 (2.7%)		
Focal to bilateral tonic–clonic seizures	38 (36.2%)	20 (36.4%)	12 (32.4%)		
Status epilepticus	5 (4.8%)	0	4 (10.8%)		
Classification of seizures by site of origin				2.494	0.646
Temporal lobe	43 (40.9%)	16 (20%)	12 (23.5%)		
Frontal lobe	45 (42.9%)	23 (28.7%)	12 (23.5%)		
Parietal lobe	14 (13.3%)	4 (5%)	6 (11.8%)		
Occipital lobe	27 (25.7%)	12 (15%)	7 (13.7%)		
Central region	12 (11.4%)	8 (10%)	4 (7.8%)		
Unknown	20 (19.0%)	17 (21.3%)	10 (19.6%)		
Classification of the causes of epilepsy				6.491	0.165
Structural	37 (35.2%)	18 (32.7%)	14 (37.8%)		
Genetic	34 (32.4%)	14 (25.5%)	14 (37.8%)		
Infectious	2 (1.9%)	1 (1.8%)	1 (2.7%)		
Metabolic	1 (1.0%)	0	1 (2.7%)		
Unknown	31 (29.5%)	22 (40%)	7 (18.9%)		
Epilepsy syndrome				4.180	0.124
Self‐limited familial infantile epilepsy, SeLIE	4 (3.8%)	4 (7.3%)	0		
Generalized epilepsy with febrile seizure, GEFS+	1 (1.0%)	0	1 (2.7%)		
Cranial MRI abnormalities	49 (46.7%)	21 (38.2%)	22 (59.4%)	19.153	0.024
Brain softening, atrophy	13 (12.4%)	7 (12.7%)	5 (13.5%)		
Neuronal migration disorder	9 (8.6%)	3 (5.5%)	4 (10.8%)		
Hippocampal sclerosis	2 (1.9%)	0	2 (5.4%)		
Electroencephalogram findings				1.885	0.757
Basic background rhythm slowing	10 (9.5%)	6 (10.9%)	3 (8.1%)		
Focal discharge	46 (43.8%)	22 (40%)	19 (51.4%)		
Multifocal discharges	29 (27.6%)	16 (29.1%)	9 (24.3%)		
Genetic abnormality	42 (40%)	15 (27.2%)	19 (51.3%)	0.411	0.522
*SCN1A* gene variation	7 (6.7%)	1 (1.8%)	4 (10.8%)		
*SCN8A* gene variation	4 (3.8%)	4 (7.3%)	0		
*DOCK6* gene variation	3 (2.8%)	1 (1.8%)	2 (5.4%)		
*mTOR* gene variation	2 (1.9%)	1 (1.8%)	1 (2.7%)		
*PRRT2* gene variation	2 (1.9%)	1 (1.8%)	1 (2.7%)		
Gene copy number variations	5 (4.8%)	1 (1.8%)	3 (8.1%)		
Other gene variations^a^	19 (18.1%)	6 (10.9%)	8 (21.6%)		
Number of ASMs utilized at baseline				7.579	0.108
1	58 (55.2%)	36 (65.5%)	17 (45.9%)		
2	26 (24.8%)	10 (18.2%)	9 (24.3%)		
3	15 (14.3%)	8 (14.5%)	6 (16.2%)		
4	4 (3.8%)	1 (1.8%)	3 (8.1%)		
5	2 (1.9%)	0	2 (5.4%)		
LCM dose, mg/(kg·d)	7.14 (4.08,10)	7.69 (4,10)	6.43 ± 4.89	−1.104	0.270
The order of LCM introduction				12.750	0.026
1	61 (58.7%)	39 (70.9%)	15 (40.5%)		
2	28 (26.7%)	12 (21.8%)	13 (35.1%)		
3	7 (6.7%)	2 (3.6%)	2 (5.4%)		
4	4 (3.8%)	1 (1.8%)	3 (8.1%)		
5	3 (2.9%)	0	3 (8.1%)		
6	2 (1.9%)	1 (1.8%	1 (2.7%)		
Diagnosis of drug‐resistant epilepsy	39 (37.1%)	19 (34.5%)	20 (54.1%)	3.447	0.063

Abbreviations: ASM, antiseizure medication; DQ, developmental quotient; GDS, Gesell Developmental Schedules; LCM, lacosamide; MRI, magnetic resonance imaging.

^a^ (1) The study population included 19 cases of other variants, including one case each of *APT1A2*, *WDR45*, *SNPRN*, *PIGA*, *CLCN2*, *ATP1A3*, *DEPDC5*, *KCNA2*, *CNC*, *SHANK3*, *SLC9A6*, *KCNQ2*, *MAP2K2*, *POLG*, and *PCDH19* gene mutations, and four cases of unknown variants. (2) Six cases of other variants were included in the clinically effective group: one case each of *PIGA*, *CLCN2*, *KCNA2*, and *SHANK3* gene mutations, and two cases of unknown variants. (3) Eight cases of other variants were included in the ineffective group: one case each of *APT1A2*, *ATP1A3*, KCNQ2, *MAP2K2*, and *PCDH19* gene mutations.

### 3.2 Efficacy

**FIGURE 1:**
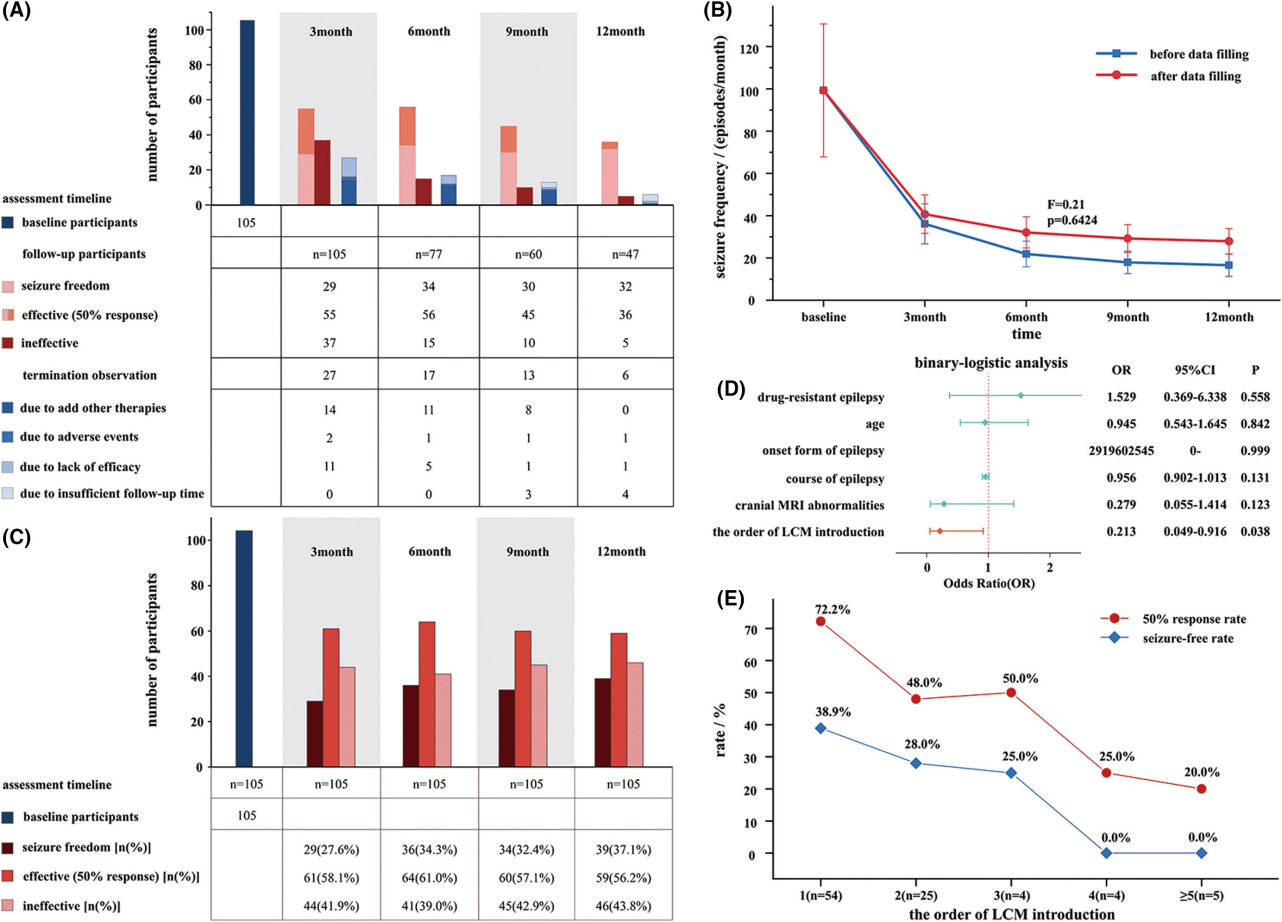
Analysis of the short‐term (3 month) efficacy of adjunctive lacosamide (LCM) therapy for focal seizures in younger children aged ≥1 month to ≤4 years. (A) Analysis of the follow‐up of LCM addition at different times; (B) analysis of the mean seizure frequency (“seizures/month”) in children at different time points after LCM addition before and after data filling; (C) analysis of the efficacy of adjunctive LCM therapy for focal seizures at different time points (after data filling, *n* = 105); (D) multifactorial analysis of the factors affecting the short‐term efficacy of adjunctive LCM therapy for focal seizures in younger children (drug‐resistant epilepsy [no or yes], age [≥1 month–≤4 years], onset form of epilepsy [SE or others], course of epilepsy, cranial magnetic resonance imaging (MRI) abnormalities [normal vs abnormal], and the order of LCM introduction [1 type vs ≥2 types]); (E) analysis of the 50% response rate and seizure‐free rate of adjunctive LCM therapy for focal epilepsy in younger children with different order of LCM introduction.

The children were followed up at 3, 6, 9, and 12 months (Figure 1A). Different strategies were used to fill the data, and MMRM models were constructed before and after data filling. In the evaluation of the fitting effect, the −2* residual log likelihood value (logistic regression value) was 10770.7; the time variable significantly affected the patients' seizure frequency (*F* = 24.26, *p* < 0.0001); and data filling (*F* = 0.21, *p* = 0.6434) and the interaction variable (*F* = 0.58, *p* = 0.58) did not affect the patients' seizure frequency (*p* > 0.05) (Figure 1B). Therefore, we used the post‐fill data to calculate the efficiency of adjunctive LCM therapy for different time periods (Figure 1C), and the 50% response rates at 3, 6, 9, and 12 months were 58.1%, 61.0%, 57.1%, and 56.2%, respectively.

The neurodevelopmental levels of 41 children treated with LCM for ≥1 year were evaluated before and after treatment (Table 2). The number of children with DQ ≥75 in each domain (social, adaptive, gross motor, fine motor, and language domains) before and after adjunctive LCM treatment is shown in Table 2. The levels for each domain were found to increase after 1 year of LCM treatment in comparison with the pretreatment levels (*p* < 0.05).

**TABLE 2:** Gesell Developmental Schedules (GDS) developmental quotient(DQ) scores before and after 1 year of adjunctive lacosamide therapy (*n* = 41).

	Social domain	Adaptive domain	Gross motor domain	Fine motor domain	Language domain
Baseline					
DQ scores ≥75, *n*[%]	18 (43.9%)	21 (51.2%)	20 (48.8%)	20 (48.8%)	17 (41.5%)
Mean DQ scores for normal children (DQ scores ≥75)	87.50 (80.75, 89.00)	89.00 (80.45, 90.50)	90.00 (84.28, 91.00)	89.90 (80.78, 90.75)	86.19 ± 1.46
Mean DQ scores for all children	69 (48.70,86.50)^▲^	75.00 (48.45,89.00)^▲^	71.20 (52.10,90.00)^▲^	70.30 (50.00,89.80)^▲^	68.50 (49.90,85.00)^▲▲^
After 1 year					
DQ scores ≥75, *n*[%]	19 (46.3%)	20 (48.8%)	21 (51.2%)	20 (48.8%)	19 (46.3%)
Mean DQ scores for normal children (DQ scores ≥75)	88.00 (83.00, 90.00)	88.12 ± 1.22	90.00 (83.00, 93.00)	87.87 ± 1.05	86.48 ± 1.38
Mean DQ scores for all children	74.00 (50.05,88.00)^*●^	74.90 (51.05,89.95)^*●^	75.10 (52.05,92.00)^*●^	74.80 (51.25,89.45)^*●^	71.90 (51.50,87.00)^*●^

*Note*: (1) Compared with baseline mean DQ scores for all children in the same domain, *p < 0.001. (2) Compared with baseline mean DQ scores for normal children (DQ scores ≥75) in the same domain, ^▲^ *p* < 0.01, ^▲▲^ *p* < 0.001. (3) Compared with mean DQ scores for normal children (DQ scores ≥75) after 1 year of adjunctive lacosamide therapy in the same domain, ^●^ *p* < 0.01.

Abbreviation: DQ, developmental quotient.

### 3.3 Analysis of risk factors affecting the short‐term efficacy of adjunctive LCM therapy

Univariate analysis indicated that mean seizure duration (*p* = 0.001), seizure type (*p* = 0.049), cranial MRI abnormalities (*p* = 0.024), and the order of LCM introduction (*p* = 0.026) may be risk factors affecting the short‐term efficacy of LCM (Table 1). All factors with *p* < 0.1 were included in the binary logistic regression analysis, and the order of LCM introduction was found to be an independent risk factor affecting short‐term efficacy (*p* = 0.038; Figure 1D). The use of LCM as the first adjunctive therapy to treat children (aged 1 month–4 years) with focal seizures yielded a 50% response rate of 72.2% and a seizure‐free rate of 38.9%; in contrast, in patients previously receiving 2, 3, 4, and 5 or more ineffective ASMs treatments, adjunctive LCM therapy had 50% response rates of 48.0%, 50.0%, 25.0%, and 20.0%, and seizure‐free rates of 28.0%, 25.0%, 0.0%, and 0.0%, respectively (Figure 1E).

### 3.4 Drug retention rate

**FIGURE 2:**
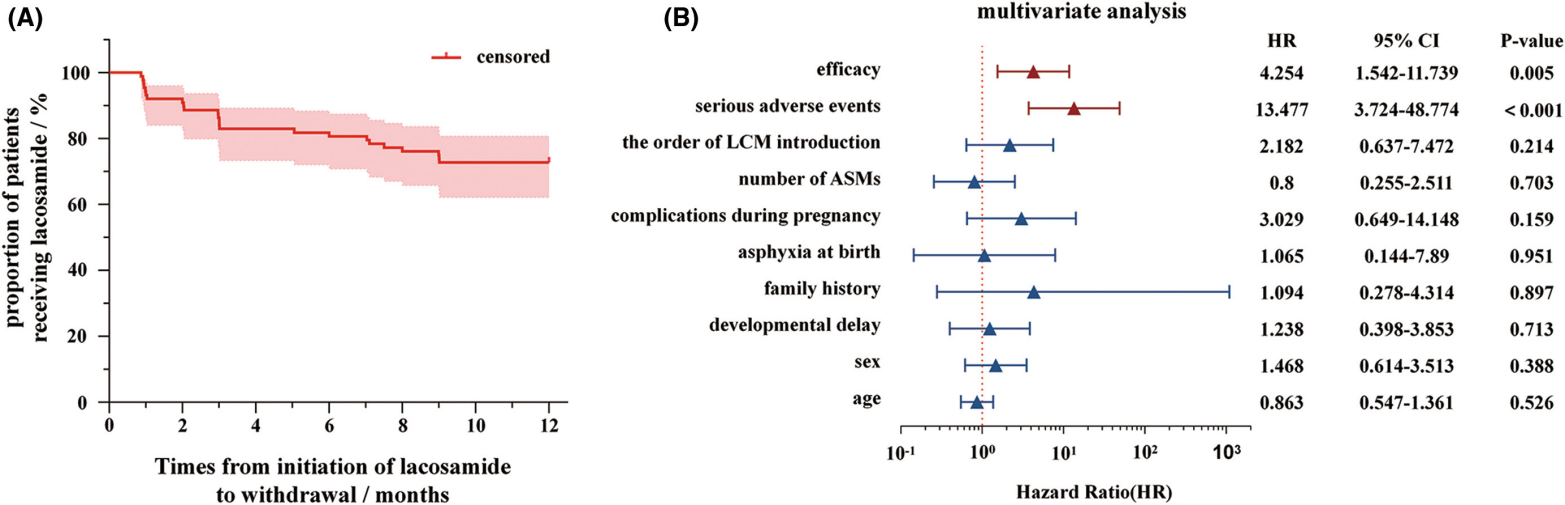
Analysis of drug retention in younger children with focal epilepsy who were treated with lacosamide (LCM) adjunctive therapy. (A) K‐M analysis of drug retention at 1 year of LCM addition (*n* = 88); (B) Cox regression analysis of factors affecting drug retention in patients receiving adjunctive LCM therapy (age [≥1 month–≤ 4 years], sex [male vs female], developmental delay [no vs yes], family history [normal vs abnormal], asphyxia at birth [no vs yes], complications during pregnancy [no vs yes], number of anti‐seizure medications (ASMs) used at baseline [1 type vs ≥2 types], the order of LCM introduction [1 type vs ≥2 types], serious adverse events [no vs yes], efficacy [effective vs ineffective]).

Drug retention was analyzed in 88 children who were observed for more than 1 year, and the retention rates after 3, 6, 9, and 12 months of adjunctive LCM therapy were 88.6% (78/88), 81.8% (72/88), 76.1% (67/88), and 72.7% (64/88), respectively, with the 95% confidence interval (CI) for drug retention after 1 year of adjunctive LCM therapy ranging from 62.1% to 80.8% (Figure 2A). Multifactorial COX regression analysis showed that lack of efficacy (*p* = 0.005) and/or occurrence of serious adverse events (*p* < 0.001) were independent risk factors for drug retention (Figure 2B).

### 3.5 Safety and tolerability

Safety and tolerability was assessed in children who continued to use LCM after 3, 6, 9, and 12 months of treatment, and adverse events to LCM at these time points were reported by 13 (12.4%, 13/105), 9 (9.9%, 9/91), 7 (9.0%, 7/78), and 6 (9.1%, 6/66) parents or carers of these children, respectively (Table 3). During the follow‐up period, a total of 13 (12.4%, 13/105) parents or carers of these children reported adverse events, including drowsiness (6/13), irritability (4/13), gastrointestinal discomfort (vomiting, abdominal pain, and nausea) (4/13), dizziness (1/13), ataxia (1/13), and neck itching with eczema (1/13). Five patients (4.7%, 5/105) withdrew from adjunctive LCM therapy due to adverse events, including vomiting drowsiness, ataxia (1/5), neck itching with eczema (1/5), irritability (2/5), and gastrointestinal discomfort (1/5). None of the children experienced cardiac, hepatic or renal impairment, or hematological adverse events.

**TABLE 3:** Adverse events related to adjunctive lacosamide therapy at different time points.

	3 months (*n* = 105)	6 months (*n* = 91)	9 months (*n* = 78)	12 months (*n* = 66)
Incidence of adverse events	13(12.4%)	9(9.9%)	7(9.0%)	6(9.1%)
Types of adverse events	Dizziness (1) Drowsiness (4) Gastrointestinal discomfort (3) Drowsiness, irritability (1) Irritability (2) Vomiting drowsiness, ataxia (1, withdrawn) Neck itching with eczema (1, withdrawn)	Dizziness (1) Drowsiness (2) Gastrointestinal discomfort (2) Drowsiness, irritability (1) Irritability (3, 1 withdrawn)	Dizziness (1) Drowsiness (2) Gastrointestinal discomfort (1) Drowsiness, irritability 1) Irritability (2, 1 withdrawn)	Dizziness (1) Drowsiness (2) Gastrointestinal discomfort (1, withdrawn) Drowsiness, irritability (1) Irritability (1)

## 4 DISCUSSION

The pediatric population is a vulnerable group, and clinical research on this population is associated with multiple challenges such as a stricter ethical review, enrollment difficulties, and poor compliance in clinical trials. In comparison with a traditional clinical trial, RWS involves a research environment and intervention measures that are closer to the actual diagnosis and treatment situation, making them more widely applicable. We could find no previous reports of large‐sample RWS has evaluated the efficacy, safety, and tolerability of adjunctive LCM therapy in the treatment of young children aged ≥1 month to ≤4 years with focal seizures. This study suggests that LCM is effective, safe, and well‐tolerated as an adjunctive therapy in young Chinese children.

A multicenter, randomized, double‐blind, placebo‐controlled clinical trial (SP0969) showed that the addition of LCM in children with focal epilepsy aged 4–17 years could reduce the frequency of seizures by more than 50% in more than half of the patients. In the maintenance period, the proportion of seizure‐free days in the children receiving LCM treatment was 0.71, which was significantly higher than that in the placebo (0.66) group (least squares mean treatment difference 0.07 (95% CI 0.029–0.115, *p* = 0.0011)).^15^ In another study, 60 Chinese children aged 4–17 years with focal epilepsy received adjunctive LCM therapy, and their 50% and 75% response rates from baseline to the last follow‐up were 40.0% (24/60) and 28.3% (17/60), respectively.^28^ The study by Driessen et al. showed that the effective rates (50% response rates) of LCM in children with drug‐resistant epilepsy were 60.5% (46/76), 67.9% (36/53), and 71.4% (30/42) at 3, 12, and 24 months, respectively.^29^ Similarly, a RWS of children showed that the 50% response rates were 47.6% (50/105), 39.2% (38/97), and 31.9% (23/72) at 3, 6, and 12 months of adjunctive LCM therapy, respectively.^30^ In our study, the 50% response rates at 3, 6, 9, and 12 months of adjunctive LCM therapy in 105 young children with focal seizures were 58.1%, 61.0%, 57.1%, and 56.2%, respectively. The results of these studies suggest that adjunctive LCM therapy can effectively reduce the frequency of seizures.

LCM also shows no clinically significant drug–drug interactions and may improve children's cognition,^31^ as well as executive function and verbal memory.^32^ Studies by Pasha et al.^33^ and Farkas et al.^15^ showed that Connor's behavior rating scale (clinical index) and CBCL Child Behavior Scale scores significantly improved after LCM treatment. Our study confirmed that after the addition of LCM treatment, the children's development levels for each domain improved in comparison with those before treatment. This may be due to normal development in young children, the positive effect of LCM on the neurodevelopmental level, or a combination of both these factors.^34^

Binary logistic regression analysis confirmed that the order of LCM introduction was an independent risk factor for short‐term efficacy. In the present study, the 50% response rate in cases in which LCM was the first adjunctive therapy for the treatment of focal epilepsy was 72.2%, and the seizure‐free rate was 38.9%. They were higher than that of the second and later addition added treatment. These findings are consistent with those reported by Zhao et al.,^35^ who showed that earlier introduction of LCM improved the likelihood of relieving epilepsy. Although the likelihood of seizure relief with each newly tried ASM decreases with the number of previously failed drugs, drug‐resistant epilepsy (following the ILAE definition which is failure of adequate trials of two tolerated, appropriately chosen and used anti‐seizure medication schedules (whether as monotherapies or in combination) to achieve sustained seizure freedom)^22^ is not equivalent to non‐response (i.e., seizure freedom was not achieved) to any drug treatment.^36^

Drug retention is an important determinant of long‐term effectiveness. In the study by Driessen et al., the retention rates of LCM therapy at 3, 12, and 24 months were 89.9% (*n* = 71), 68.4% (*n* = 54), and 54.4% (*n* = 43), respectively. The main reason for discontinuation was lack of efficacy, followed by adverse events.^29^ In a retrospective observational study, 65% of children continued treatment with LCM for 12 months or more. Children with any type of epilepsy showed similar retention rates. The drug was discontinued mainly because of a lack of efficacy. Only 18% of children discontinued LCM because of adverse events.^37^ The one‐year drug retention rate with LCM for epilepsy in Japanese adults was 73% in the study by Siming Chen et al.^38^ Similar to the results of the present study, the drug retention rates of short‐term (3 months) and long‐term (12 months) adjunctive lacosamide therapy in a previous study were 88.6% and 72.7%, respectively. Thus, lack of efficacy and serious adverse events were independent risk factors for drug retention.

Several studies have demonstrated that LCM treatment in children with focal seizures has a favorable safety profile and is well‐tolerated, showing common neurological and gastrointestinal system‐related adverse events and no other safety signals.^29^, ^37^ A meta‐analysis showed that adverse events occurred in 31.5% of pediatric patients, with the most common adverse events being drowsiness (15.0%), dizziness (9.9%), and somnolence (8.3%).^39^ The most common adverse events in this study were drowsiness, gastrointestinal discomfort, and irritability, with an adverse event rate of 12.4%, and 4.7% of the children withdrew due to the occurrence of serious adverse events. Conventional sodium channel blockers containing an aromatic ring structure have been shown to cause a higher percentage of skin sensitization reactions, while studies in Chinese and Japanese epileptic adults (16–70 years) treated with LCM have shown a low incidence of rash‐related adverse events (2.5%), all of which were mild or moderate.^40^ Moreover, the occurrence of rash was not reported in several RWS on LCM.^41^, ^42^ The present study observed one (1/105, 0.94%) mild drug‐related skin adverse events, suggesting that the molecular structure and mechanism of action of LCM are different from those of traditional sodium channel blockers and that it causes a low incidence of skin allergic reactions. In this study of 105 Chinese children, it had a favorable safety and tolerability profile.

In the future, prospective and longer follow‐up studies can reduce potential bias, further clarify the long‐term efficacy, safety, and tolerability of adjunctive LCM therapy in younger Chinese children with focal seizures, and provide clinical evidence for adjunctive LCM therapy in patients older than 1 month.

## 5 CONCLUSION

LCM was effective, safe, and well‐tolerated as an adjunctive therapy in young Chinese children (1 month to 4 years) with focal seizures in this study.

## AUTHOR CONTRIBUTIONS

Substantial contributions to the conception or design of the work: Yue Hu, Lu Yang. Drafting the work or revising it critically for important intellectual content: All authors. Final approval of the version to be published: Lu Yang, Yuhang Liu, Yue Hu. Agreement to be accountable for all aspects of the work in ensuring that questions related to the accuracy or integrity of any part of the work are appropriately investigated and resolved: All authors.

## FUNDING INFORMATION

No funding was provided for this study.

## CONFLICT OF INTEREST STATEMENT

We have no conflicts of interest to disclose.

## Data Availability

The datasets generated during and/or analyzed during the current study are not publicly available. However, anonymized data can be sent to the corresponding author by reasonable requests.
